# A Comparative Study of the Motivations to Teach Chinese Between Native and Non-native Pre-service CSL/CFL Teachers

**DOI:** 10.3389/fpsyg.2021.703987

**Published:** 2021-08-17

**Authors:** Ling Gu, Binglong Wang, Haiwei Zhang

**Affiliations:** College of International Education, Minzu University of China, Beijing, China

**Keywords:** language teacher motivation, native speaker teacher, non-native speaker teacher, Chinese language teacher, Chinese as a second/foreign language

## Abstract

The present study compared the motivations to teach Chinese between native and nonnative pre-service teachers of Chinese as a second/foreign language (CSL/CFL). The participants included 325 native and 325 non-native Chinese-speaking pre-service CSL/CFL teachers registered in the Masters in Teaching Chinese to Speakers of Other Languages (MTCSOL) programs; the teachers were asked to complete a 24-item questionnaire. Two major findings emerged. First, a similar six-factor teacher motivation was observed for both the native and non-native teachers. Second, the two groups showed non-significant differences in their ratings of the importance of cross-cultural value, intrinsic value, altruistic value, and fallback career choice as types of motivation but differed significantly in their ratings of extrinsic value and social influence. These results highlight the differences and similarities in the motivation of the second language teacher and offer insights into the variables at different levels that might influence the motivation of the second language teacher. Teacher motivation is advised to be taken into account in the training and administration of CSL/CFL teachers to alleviate the problems of teacher shortage outside China.

## Introduction

Teacher motivation is a key topic in educational psychology and is generally defined as the psychological motives that drive an individual to choose teaching as a career. The importance of teacher motivation for various aspects of teaching and learning has been widely recognized in the field of general education (Dörnyei and Ushioda, 2011; Richardson et al., 2014; Han and Yin, 2016; Hiver et al., 2018; Kissau et al., 2019a), such as the dropout rate of student teachers (Jungert et al., 2014), career decisions (Watt and Richardson, 2007, 2008; Kissau et al., 2019a), teaching performance (Irnidayanti et al., 2020) and learning motivation of students (Wild et al., 1997; Atkinson, 2000; Roth et al., 2007; Radel et al., 2010; Jodaei et al., 2018). Since the1990s, in the field of second language education (Pennington, 1995), a considerable body of literature has explored the motivation to become a second language (L2) teacher. One potential reason for carrying out such research relates to the shortage of L2 teachers, a problem commonly observed in many countries around the world (Swanson, 2010, 2012; Swanson and Huff, 2010; Swanson and Mason, 2017; Kissau et al., 2019a). Previous research on L2 teacher motivation mainly focused on English language teachers, with insufficient attention paid to teachers of less commonly taught languages, such as Chinese.

The growing importance of learning Chinese as a second/foreign language (CSL/CFL) has been commonly acknowledged around the world. By December 2018, 492,185 foreign students from 196 countries studied in 1,004 universities or colleges in China (Ministry of Education of the People's Republic of China, 2019). By December 2019, 550 Confucius Institutes and 1,172 Confucius Classrooms have been founded in 162 countries; more than 30,000 elementary and middle schools and more than 4,000 universities offered Chinese language courses and more than 25 million people were learning Chinese^1^. Such a growth of Chinese language education leads to a shortage of qualified CSL/CFL teachers in many countries and areas outside China to some extent.

To help solve this problem, first, the Chinese government has established Masters in Teaching Chinese to Speakers of Other Languages (MTCSOL) programs at 149 universities by December 2020^2^ and has funded about 105,000 native CSL/CFL teachers to teach abroad from 2004 to 2018^3^. In addition, the Center for Language Education and Cooperation (CLEC, previously known as *Hanban*) and China Scholarship Council have been providing scholarships for non-native Chinese speakers to study MTCSOL programs in China, aiming to train Chinese language teachers for local contexts outside China. However, a considerable proportion of native and non-native preservice teachers have ultimately not worked as CSL/CFL teachers after completing the program (Liu, 2016; Li, 2017), a common problem that has been observed for teaching in other languages (Swanson and Huff, 2010).

Second, along with a noteworthy growth in researching Chinese language education (Ma et al., 2017; Gong et al., 2018b, 2020a,c; Ke, 2020; Lü, 2020), researchers have carried out studies on the professional development of CSL/CFL teachers (such as Moloney, 2013; Moloney and Xu, 2015; Ma and Gao, 2017; Gong et al., 2018a; Wang and Bale, 2019; Yang, 2019; Zhang et al., 2020), focusing on topics, such as teacher cognition, teacher knowledge, and teacher education programs. However, empirical studies on CSL/CFL teachers are relatively few (Ma et al., 2017; Gong et al., 2020a) and existing research mainly concentrated on the in-service native CSL/CFL teachers, paying less attention to the comparison between the preservice native and non-native CSL/CFL teachers.

Compared with the rapid development of Chinese language education and MTCSOL programs, our understanding of the reasons why native and non-native preservice CSL/CFL teachers choose to enter Chinese teaching profession is still relatively inadequate. Comparing the two groups, teacher motivation is crucial. First, the two groups are critical for solving the problem of CSL/CFL teacher shortage outside China because, after completing the master's program, most non-native preservice CSL/CFL teachers return to their home countries and many native preservice CSL/CFL teachers tend to teach Chinese abroad. Thus, teacher motivation might determine the decision of the two groups to continue CSL/CFL teaching or not. Second, most MTCSOL programs administer a split-class model, where the native and non-native groups study separately, based on the assumed differences between the two groups in terms of Chinese language proficiency, professional knowledge, and teacher cognition. However, it is not clear whether the two groups have different or similar teacher motivations, which might be helpful for designing a tailor-made MTCSOL program. Furthermore, considering common concerns of the researchers over teacher motivation across different contexts (Watt and Richardson, 2012) and limited research on comparing the native and non-native speaker and the motivation of L2 teachers, this study might be of interest and value for researchers and practitioners in other L2s and in general education. Therefore, the present study aims to investigate the similarities and differences in teacher motivation between the native and non-native pre-service CSL/CFL teachers, also as an echo to the call of the researchers for gaining more attention to empirical studies on CSL/CFL teachers (Ma et al., 2017; Gong et al., 2020a).

## Literature Review

### Motivation to Be a Teacher

Studies on the motivation to be a teacher in general education have been conducted using various frameworks, such as self-determination theory (Deci and Ryan, 1985), achievement-goal theory (Elliot, 2005), and expectancy-value theory (Eccles, 2009). Both quantitative and qualitative methods have been conducted, and one of the most popular methods is questionnaire surveys. Watt and Richardson (2007) developed a reliable and valid tool, the factors influencing teaching choice (FIT-Choice) Scale, to investigate the types of teacher motivation in general education. This questionnaire has been successfully applied in different contexts (Berger and D'Ascoli, 2012; Fokkens-Bruinsma and Canrinus, 2012; Jugović et al., 2012; Kilinç et al., 2012; König and Rothland, 2012; Lin et al., 2012; Watt and Richardson, 2012) and resulted in some consensus on the types of teacher motivation, largely aligning with the results reported in the previous studies (Brookhart and Freeman, 1992; Kyriacou et al., 1999; Kyriacou and Coulthard, 2000; Dörnyei and Ushioda, 2011; Fokkens-Bruinsma and Canrinus, 2014; Richardson et al., 2014).

First, studies have generally found that intrinsic value is a major type of teacher motivation. Interest in teaching or the subject and beliefs about the teaching abilities of an individual are inspiring motives for an individual to work as a teacher. The second type of teacher motivation relates to altruistic or social values, such as making contributions to social justice and shaping the lives of the younger generation. The third type concerns extrinsic value, i.e., perceptions of the teachers on the extrinsic welfare offered by teaching, such as the social status, job security, and job transferability of the teachers. Fourth, although the career choices of some teachers are related to encouragement from their family members or friends, they tend to show weak fallback career tendencies. In general, the decision of an individual to work as a teacher is mainly based on her/his own choice, rather than being based on the social influence from others or a view of teaching as a stepping stone to another career.

### Motivation to Be a Second Language Teacher

Similar to the results reported for general education, the motivations of L2 teachers have been found to be significant for their career satisfaction (Kassabgy et al., 2001; Kissau et al., 2019a). However, research on the motivation of an L2 teacher is scarce (Dörnyei and Ushioda, 2011; Hastings, 2012), similar to L2 motivation research lying outside the mainstream L2 acquisition research (Ellis, 2008). The L2 teachers are often recruited to be participants in research on teacher motivation in general education (Kyriacou and Coulthard, 2000). However, the uniqueness of L2 teachers has been widely acknowledged (Hammadou and Bernhardt, 1987; Borg, 2006; Dörnyei and Ushioda, 2011), such as the nature of the subject, teacher–learner relationships, and the differences between native and non-native speakers. Borg claimed that “being a foreign language teacher is in many ways unique within the profession of teaching. Becoming a foreign language teacher, too, is a different process from that which other future teachers experience” (p. 5). Thus, researching the motivation of L2 teachers might deepen our understanding of the differences and similarities between L2 teachers and their counterparts in general education, aligning with the call of Watt and Richardson (2012) for examining the influence of subject specialization on teacher motivation.

Studies on L2 teacher motivation have focused on both native and non-native L2 teachers and both in-service and preservice teachers in countries, such as China (Zhao, 2008; Shih, 2016; Zhang et al., 2020), Morocco (Kyriacou and Benmansour, 1999), Greece (Karavas, 2010), Turkey (Erkaya, 2013), the UK (Barnes, 2005), and the USA (Kissau et al., 2019a,b). Most studies have concentrated on teachers teaching English as a second language (ESL) (Pennington, 1995; Kyriacou and Benmansour, 1999; Kassabgy et al., 2001; Zhao, 2008; Karavas, 2010; Erkaya, 2013; Shih, 2016; Kissau et al., 2019a,b), with less attention given to other less commonly taught languages, such as Chinese (Zhu and Qian, 2015; Zhang et al., 2020).

Some common findings have emerged from prior research on L2 teacher motivation (Table A1). The top-rated types of motivation include intrinsic values, such as love and enjoyment of the target language and culture, confidence in teaching abilities, love of teaching, and working with children/adolescents, and altruistic values, such as making social contributions and nurturing students to learn and succeed. Extrinsic value, such as job transferability, improved language proficiency, job security, and work–family balance, was reported in some developing countries, such as China (Zhao, 2008; Shih, 2016) and Morocco (Kyriacou and Benmansour, 1999). Social influence, such as family approval and fallback career choice, has been the least mentioned. According to the findings summarized here and those in section Motivation to Be a Teacher, it seems that L2 teachers and their counterparts in general education have certain similar types of motivations to some extent, such as intrinsic values and altruistic values.

Studies revealed that pre-service CSL/CFL teachers might have a unique type of motivation related to cross-culture communication. Zhu and Qian (2015) reported that learning about foreign cultures ranked sixth among the reasons for student teachers to work as volunteer Chinese language teachers. Zhang et al. (2020) further observed the unique existence of the cross-cultural value of teaching, such as working with foreigners or interest in cross-cultural communications and exchanges, among native pre-service CSL/CFL teachers. The findings on cross-cultural value extracted as an independent factor suggest the uniqueness of L2 teacher motivation. However, whether cross-cultural value drives non-native preservice CSL/CFL teachers to enter teaching is still unclear.

Native and non-native L2 teachers might differ in the types and ratings of teacher motivation. For instance, Shih (2016) found that local Taiwanese teachers did not mention making a social contribution as a type of motivation to be a teacher, yet some native English teachers did. Kissau et al. (2019b) compared the ratings of teacher motivation between ESL teachers in the US (native, *n* = 54), Germany (non-native, *n* = 233), and China (non-native, *n* = 116) using the FIT-Choice scale. The US group rated intrinsic value, social contribution, and cultural connection higher than the German or Chinese group. However, the US and German groups did not differ significantly in their ratings of work with children/adolescents, job security, or social influence. Considering the limited number of participants and the unbalanced native and non-native samples in previous studies, these results should be interpreted with caution. Therefore, a systematic study investigating the similarities and differences in teacher motivation between native and non-native L2 teachers is needed.

Researchers have conducted studies on the differences and similarities between native and non-native L2 teachers from different perspectives (Llurda, 2005; Braine, 2009; Huang, 2017; Martínez Agudo, 2017). Most previous studies have been carried out among ESL teachers and have focused on topics, such as teacher education in ESL settings, as well as the advantages and disadvantages that native and non-native L2 teachers experience (Moussu and Llurda, 2008). In terms of CSL/CFL teachers, several researchers have explored professional identity, perceptions of learners regarding the teaching models of native and non-native CSL/CFL teachers and teaching pedagogy (Burns, 2014; Sung and Poole, 2016; Goh, 2017; Zhang and Wang, 2017; Bo, 2018; Zhang and Zhang, 2018). However, the existing research has paid little attention to the motivations underlying the career choices of native and non- native pre-service CSL/CFL teachers.

### The Present Study

As reviewed above, researchers have realized the increasing significance of motivation for L2 teacher education and the development of theoretical frameworks concerning teacher motivation. As Hiver et al. (2018) noted, “the domain of language teacher motivation is well positioned to become a richer field of inquiry because of its crossover appeal” (p. 17). However, insufficient studies have explored L2 teacher motivation and there are still some research gaps to be filled. One main gap is that no research has systematically compared the motivations of native and non-native pre-service L2 teachers. Previous studies about teacher motivation have concentrated on the English language, which might limit the generalization of the findings with other languages. Meanwhile, the increasing growth of CSL/CFL education and the shortage of CSL/CFL teachers call for more research on teacher motivation among the native and non-native preservice CSL/CFL teachers. More specifically, the types of teacher motivation and the ratings in each type of motivation are still not clear among the native and non-native groups. Therefore, to fill this gap, the present study compared the reasons underlying preservice career choices of L2 teachers among native and non-native speakers of Chinese. To be specific, the present study addressed the following questions:

RQ1: What are the differences and similarities in the types of teacher motivation between native and non-native preservice CSL/CFL teachers?

RQ2: What are the differences and similarities in the ratings of each type of teacher motivation between native and non-native preservice CSL/CFL teachers?

## Methodology

### Participants

In the present study, the target participants were preservice CSL/CFL teachers from MTCSOL programs in China. We are aware that stratified sampling is powerful in recruiting representative samples from the population (Neyman, 1992). However, stratified sampling is difficult for administration in the present study due to insufficient data about the characteristics of the preservice CSL/CFL teachers in MTCSOL programs. The participants were recruited using the snowball sampling method. We first sent the questionnaire to familiar CSL/CFL teacher educators at different universities, who then forwarded the questionnaire to the preservice CSL/CFL teachers they could access. The teacher educators and preservice CSL/CFL teachers were requested to further forward the questionnaire to other target participants. All participants were given an online version of the informed consent form before they filled in the questionnaire, informing them of the aim and the tasks, as well as how their personal information would be protected.

A total of 349 non-native CSL/CFL teachers were recruited from 20 universities. Twenty-four participants were excluded due to their insufficient Chinese proficiency (below HSK level 5)^4^ or a large amount of time spent completing the questionnaire (more than 30 min). Therefore, 325 participants were retained for the final analysis (mean age = 25.5 years, SD = 3.33, MIN = 20, MAX = 38), including 245 women and 80 men. Most of the participants came from Asia (*n* = 265), and the others were from Africa (*n* = 29), Europe (*n* = 28), and South America (*n* = 23). All the non-native participants were at an advanced Chinese level, with 133 participants passing HSK level 5 and 192 participants passing HSK level 6. Different from the non-native samples, the native samples were selected from a large-scale study carried out by Zhang et al. (2020). To minimize the influence of the sample size on the research results between the native and non-native groups, 325 native participants were randomly chosen out of the total pool (*n* = 411). The native group included MTCSOL students from 17 universities in Mainland China, including 294 women and 22 men (9 unreported). There were 253 participants aged between 20 and 25 and 35 aged between 26 and 30 (37 unreported). All the participants came from seven geographical regions in China, accounting for 41.18% (14/34) of the provincial-level administrative divisions. The female–male ratio in the two groups demonstrated the predominance of women students in MTCSOL programs, largely representative of the population (Ma and Gao, 2017; Gong et al., 2018a).

### Instrument

The FIT-Choice scale has been successfully applied to explore teacher motivation among ESL and CSL/CFL teachers (Shih, 2016; Kissau et al., 2019a,b; Zhang et al., 2020). In the present study, the questionnaire used for non-native pre-service CSL/CFL teachers was adopted from the study by Zhang et al. (2020), who designed a questionnaire for native preservice CSL/CFL teachers based on FIT-Choice Scale and the interview results of four native Chinese speakers^5^. The original questionnaire designed by Zhang et al. (2020) included 33 items, where 24 items were retained in the final version; the 24 items were further categorized into six factors.

To facilitate the comparison between native and non-native samples, 6 factors and 24 items were retained in the non-native version. Considering the unique language and cultural backgrounds of the non-native participants, the items measuring cross-cultural value and altruistic value were slightly altered according to the results of the interviews with 10 non-native preservice CSL/CFL teachers. For instance, the altruistic value item for native CSL/CFL teachers, “Chinese teaching can help eliminate foreigners' misunderstandings of China,” was changed to “Chinese teaching can contribute to the cultural exchange between my country and China;” the cross-cultural value item for native CSL/CFL teachers, “I like socializing with foreigners,” was altered to “I like socializing with Chinese people.”

The participants were required to rate the importance of each item for their decision to enter the Chinese teaching profession on a scale ranging from 1 (*not important at all*) to 5 (*very important*). The following statement preceded all items “I chose to become a CSL/CFL teacher because…” The questionnaire was piloted with 10 non-native preservice CSL/CFL teachers to ensure that the instructions and statements were clear and understandable. The questionnaire was further modified based on the interviews with the participants in the pilot study. The items were presented in Chinese in random order. Cronbach's alpha reliability was found to be 0.88. The design of the questionnaire was as follows.

The first factor, *cross-cultural value*, included six items, such as “I like socializing with Chinese people.” The second factor, *altruistic value*, consisted of three items, such as “Chinese teaching can help my compatriots learn Chinese.” The third factor, *intrinsic value*, included five items, such as “I have good teaching skills.” The fourth factor, *extrinsic value*, comprised four items, such as “As a CSL/CFL teacher, I can have a high salary.” The fifth factor, *social influence*, had three items, such as “My friends think I should be a CSL/CFL teacher.” There were three fallback career choices in the sixth factor, e.g., “I have not found my ideal major yet.”

### Data Collection

Previous studies have shown that the results generated from online and traditional paper-and-pencil questionnaires are comparable and consistent (Gosling et al., 2004). Due to the COVID-19 pandemic, the survey was administered online using the mobile version of *Wenjuanxing* (Questionnaire Star, Chinese version of Survey Monkey). A total of 353 out of the 400 initiated online questionnaires were submitted. The questionnaire took ~15–20 min to complete. Upon completion, the participants were entered to win a small amount of cash.

### Data Analysis

To answer RQ1 about the differences and similarities in the types of motivation in the native and non-native preservice CSL/CFL teachers, factor analyses were conducted. It has been recommended to split the sample randomly in half, with the first half for exploratory factor analysis (EFA) and the second half for confirmatory factor analysis (CFA) (MacCallum et al., 1994; Fokkema and Greiff, 2017). However, the sample in each of the native and non-native groups in the present study was not large enough to be suitable for splitting randomly into half; therefore the whole sample in each group was used for factor analyses for RQ1.

To explore RQ2 about the differences and similarities in the native and non-native pre-service ratings of CSL/CFL teachers regarding each type of motivation, the one-sample *t*-tests and the MANOVA tests were carried out.

## Results

### RQ1: Differences and Similarities in the Types of Motivation Between Native and Non-native Preservice CSL/CFL Teachers

First, CFA was carried out in the two groups of CSL/CFL teachers to explore whether the data matched the structure of teacher motivation reported by Zhang et al. (2020). The results in Table 1 indicated that the six-factor model was not acceptable in the two groups of preservice teachers.

**Table 1:** Observed fit statistics values and criteria for adequacy based on confirmatory factor analysis (CFA).

**Group**	**Chi-square**	***df***	***p***	**Chi-square/*df***	**CFI**	**TLI**	**RMSEA**
Non-native	732	237	<0.001	3.09	0.84	0.81	0.08
Native	1,012	237	<0.001	4.27	0.81	0.78	0.09
Criteria			>0.05	<2	>0.90	>0.90	<0.06

*RMSEA, root mean square error of approximation; CFI, comparative fit index; TLI, Tucker-Lewis index*.

Second, considering that the CFA results were not satisfactory, EFA was utilized to further analyze the factors of teacher motivation in the two groups. The data collected from each of the two samples were suitable for factor analysis: native group, Kaiser-Meyer-Olkin (KMO) = 0.86, Bartlett's K^2^ = 4,341, *df* = 276, *p* < 0.001; non-native group, KMO = 0.88, Bartlett's K^2^ = 3,227, *df* = 276, *p* < 0.001. Principal axis factoring and oblimin rotation were chosen because the dimensions describing the structure were closely intercorrelated (Zhang et al., 2020). For factor extraction, two methods were attempted. The first was parallel analysis, and the second was the determination of a fixed number of factors to be retained (*n* = 6) based on the questionnaire design. The cut-off factor loading was 0.40 (Henson and Roberts, 2006; Osborne et al., 2008; Plonsky and Gonulal, 2015)^6^.

In the native group, EFA with parallel analysis and a fixed number of factors produced the same results. Six factors were produced with all the items retained, accounting for 55.6% of the total variance. The general results were interpretable. Most of the correlation coefficients between the six factors ranged from small to medium, indicating the validity of the use of the oblimin rotation. The fit statistics of the overall model were acceptable (Table 2). Most of the extracted factors were correlated with each other (Table 3).

**Table 2:** Observed fit statistics values and criteria for adequacy based on exploratory factor analysis (EFA).

**Group**	**Factor number**	**Chi-square**	***df***	***p***	**Chi-square/df**	**TLI**	**RMSEA**
Native	Parallel analysis	278	147	<0.001	1.89	0.94	0.05
	Fixed number	278	147	<0.001	1.89	0.94	0.05
Non-native	Parallel analysis	416	166	<0.001	2.51	0.86	0.07
	Fixed number	291	147	<0.001	1.98	0.91	0.05
Criteria				>0.05	<2	>0.90	<0.06

**Table 3:** Correlation matrix between the extracted factors (native below and non-native above).

	**1**	**2**	**3**	**4**	**5**	**6**
Intrinsic		0.53	0.45	0.38	0.45	−0.24
Altruistic	0.45		0.46	0.58	0.34	−0.12
Social influence	0.34	0.32		0.46	0.59	0.30
Cross culture	0.43	0.46	0.34		0.23	0.09
Extrinsic	0.46	0.12	0.46	0.27		0.25
Fallback-career	−0.18	−0.32	0.17	−0.06	0.16	

In the non-native group, the first attempt with parallel analysis produced five factors with 20 items retained. Although the general results were interpretable, the five factors explained only 48.7% of the total variance, and the fit measures of the overall model were not acceptable (Table 2). Therefore, a second attempt with a fixed number of factors (*n* = 6) was carried out. The second EFA retained six factors with 21 items, which accounted for 51.7% of the total variance. The results were interpretable, and the overall model was acceptable (Table 2). Most of the extracted factors were correlated with each other (Table 3).

The EFA results in the two groups are presented in Tables 4, 5. The two groups demonstrated the same factors of teacher motivation, including cross-cultural value, intrinsic value, extrinsic value, altruistic value, social influence, and fallback career choice. However, there were certain differences in the factor loadings of some items. For instance, “I am interested in learning about different cultures” and “I like teaching others to learn a foreign language,” were retained in the cross-cultural value factor in the native group, yet these two items did not load on any factor in the non-native group. “I like socializing with foreigners” was grouped under the extrinsic value factor in the native group, yet its corresponding statement, “I like socializing with Chinese people” loaded on the cross-cultural value factor in the non-native group. In contrast, “As a CSL/CFL teacher, I can have more opportunities to work abroad” was grouped under extrinsic value in the non-native group and under cross-cultural value in the native group.

**Table 4:** Summary of the factor loadings of EFA (native group).

**Item**	**Factor**	**% of variance**	**α/ω**	**1**	**2**	**3**	**4**	**5**	**6**	**Uniqueness**
Chinese teaching could allow more family time.	Cross-cultural value	11.41	0.83/0.83	0.85						0.26
I like working in an environment that involves being in contact with foreigners.				0.77						0.35
I have had pleasant communication experiences with foreigners.				0.58						0.57
As a CSL/CFL teacher, I can have more opportunities to work abroad.				0.54						0.67
I am interested in learning about different cultures.				0.46						0.71
I like teaching others to learn a foreign language.				0.46						0.56
Teaching is a career suited to my abilities.	Intrinsic value	10.69	0.84/0.84		0.64					0.49
I am interested in teaching.					0.70					0.42
I have good teaching skills.					0.69					0.36
I have the qualities of a good teacher.					0.68					0.44
I have always wanted to be a teacher.					0.53					0.50
As a CSL/CFL teacher, I can have a stable income.	Extrinsic value	9.14	0.80/0.81			0.85				0.21
As a CSL/CFL teacher, I can have a high salary.						0.81				0.36
I like socializing with foreigners.						0.54				0.67
Chinese teaching is a secure job.						0.42				0.51
My friends think I should be a CSL/CFL teacher.	Social influence	8.70	0.82/0.82				0.81			0.39
My teachers/classmates think I should to be a CSL/CFL teacher.							0.74			0.38
My family/relatives think I should be a CSL/CFL teacher.							0.67			0.36
Chinese teaching can help improve the international image of China.	Altruistic value	8.26	0.80/0.81					0.82		0.37
Chinese teaching can help communicate Chinese culture to other countries.								0.70		0.34
Chinese teaching can help eliminate foreigners' misunderstandings of China.								0.69		0.51
I have not found my ideal major yet.	Fallback-career value	7.36	0.78/0.79						0.82	0.32
I chose CSL/CFL teaching as a last-resort career.									0.75	0.45
I was unsure of what career I wanted.									0.63	0.48

**Table 5:** Summary of the factor loadings of EFA (non-native group).

**Item**	**Factor**	**% of variance**	**α/ω**	**1**	**2**	**3**	**4**	**5**	**6**	**Uniqueness**
I am interested in teaching.	Intrinsic value	13.33	0.85/0.85	0.77						0.28
Teaching is a career suited to my abilities.				0.70						0.40
I have good teaching skills.				0.70						0.43
I have always wanted to be a teacher.				0.61						0.50
I have the qualities of a good teacher.				0.55						0.61
Chinese teaching can help communicate Chinese culture to my country.	Altruistic value	8.40	0.75/0.76		0.71					0.39
Chinese teaching can help my compatriots have a better understanding of China.					0.71					0.45
Chinese teaching can help my compatriots learn Chinese.					0.50					0.55
My teachers/classmates think I should be a CSL/CFL teacher.	Social influence	8.57	0.78/0.78			0.77				0.30
My friends think I should be a CSL/CFL teacher.						0.67				0.42
My family/relatives think I should be a CSL/CFL teacher.						0.47				0.58
I have had pleasant communication experiences with Chinese people.	Cross-cultural value	7.78	0.77/0.77				0.75			0.32
I like working in an environment that involves being in contact with Chinese people.							0.73			0.48
I like socializing with Chinese people.							0.50			0.48
As a CSL/CFL teacher, I can have a stable income.	Extrinsic value	7.47	0.75/0.75					0.81		0.36
As a CSL/CFL teacher, I can have a high salary.								0.56		0.63
As a CSL/CFL teacher, I can have more opportunities to work abroad.								0.53		0.59
Chinese teaching is a secure job.								0.44		0.52
I have not found my ideal major yet.	Fallback-career value	6.15	0.69/0.70						0.74	0.45
I was unsure of what career I wanted.									0.73	0.41
I chose Chinese teaching as a last-resort career.									0.42	0.73

### RQ2: Differences and Similarities in the Ratings of Each Type of Motivation Between Native and Non-native Preservice CSL/CFL Teachers

First, the mean scores of two groups on each factor were compared with the scale midpoint of 3 (Table 6). The scores of both the groups for fallback career choice were not significantly different from 3, and the effect sizes were small. Both the samples showed similar patterns of ratings of the importance of altruistic value, cross-cultural value, and intrinsic value, and the scores were significantly higher than 3, with large or close-to-large effect sizes. However, the two groups showed opposite rating patterns for extrinsic value and social influence. In terms of extrinsic value, the score of the non-native group was significantly higher than 3, with a large effect size, while the score of the native group was lower than the midpoint, and the effect size was small. Regarding social influence, the rating was higher than 3 in the non-native group and lower than 3 in the native group, but the effect sizes were medium in both the groups.

**Table 6:** Summary of the ratings of the native and non-native groups in each factor and the results of one-sample *t*-tests.

**Group**	**Factor**	**Mean**	**SD**	***t***	***df***	***p***	**Cohen's d**
Non-native	Altruistic value	4.36	0.62	39.29	324	<0.001	2.18
	Cross-culture value	4.17	0.72	29.46	324	<0.001	1.63
	Intrinsic value	3.89	0.75	21.61	324	<0.001	1.20
	Extrinsic value	3.69	0.70	17.79	324	<0.001	0.99
	Social influence	3.35	1.00	6.37	324	<0.001	0.35
	Fallback career	2.97	0.96	−0.64	324	0.52	0.04
Native	Altruistic value	3.93	0.79	21.18	324	<0.001	1.17
	Cross-culture value	3.83	0.74	20.34	324	<0.001	1.13
	Intrinsic value	3.64	0.80	14.50	324	<0.001	0.80
	Fallback career	2.93	0.92	−1.35	324	0.18	0.07
	Extrinsic value	2.86	0.86	−2.92	324	0.004	0.16
	Social influence	2.67	0.95	−6.25	324	<0.001	0.35

Second, Multivariate analysis of variance (MANOVA) tests were carried out to explore the teacher motivation ratings of the two groups. MANOVA tests were used because the six factors were correlated (Table 3) and Type I error inflation could occur when conducting multiple independent *t*-tests (O'Brien and Kaiser, 1985; Warne, 2014). In light of previous studies (Lin et al., 2012; Watt and Richardson, 2012) and considering that altruistic value included different items in the two groups and that some factors such as cross-cultural value and extrinsic value differed in the number of items across the two groups, it was inappropriate to use the original questionnaire to compare the differences of the two groups, which might come from the samples and/or the items.

To make the items in each factor matched in the two groups, only five of the six factors (altruistic value excluded) and the items loading on the same factors in the two groups were kept (see items with # in Table A2). The shortened questionnaire with 16 items had a good reliability: native group, Cronbach's alpha = 0.82, McDonald' ω = 0.84; non-native group, Cronbach's alpha = 0.81, McDonald's ω = 0.82. The ratings of the two groups in these five factors in the shortened questionnaire showed similar patterns (Table 7) as those observed using the original questionnaires (Table 6), indicating that these questionnaires were comparable to a greater extent.

**Table 7:** Summary of the MANOVA test.

**Factor**	**Native**	**Non-native**	***F***	***df1***	***df2***	***p***	**Partial η^2^**
Cross-cultural value	3.71 (0.89)	4.08 (0.81)	30.66	1	646	<0.001	0.05
Intrinsic value	3.64 (0.80)	3.89 (0.75)	17.82	1	646	<0.001	0.03
Extrinsic value	2.92 (0.92)	3.58 (0.77)	98.85	1	646	<0.001	0.13
Social influence	2.67 (0.95)	3.35 (1.00)	80.53	1	646	<0.001	0.11
Fallback career	2.93 (0.92)	2.97 (0.96)	0.32	1	646	0.57	0.001

The results of MANOVA analyses revealed that the main effect of L1 group was significant and the effect size was large (Cohen, 1988), λ = 0.83, *F*_(5,642)_ = 27, *p* < 0.001, partial η^2^ = 0.17. Univariate tests showed significant main effects for L1 group in four factors, and the effect size ranged from small (cross-cultural value and intrinsic value) to large (extrinsic value and social influence); the main effect of L1 group on fallback career choice was not significant and the effect size was small (Table 7).

## Discussion

### Similarities in Motivation Between Native and Non-native CSL/CFL Teachers

First, the present study found similar types of motivation among both native and non-native preservice CSL/CFL teachers, including cross-cultural, intrinsic, altruistic, and extrinsic values, social influence, and fallback career choice. The findings on intrinsic, altruistic, and extrinsic values, social influence, and fallback career choice resonated with studies using the FIT-Choice scale in L2 teachers (Shih, 2016; Kissau et al., 2019a,b; Zhang et al., 2020) and general education teachers (Berger and D'Ascoli, 2012; Fokkens-Bruinsma and Canrinus, 2012; Jugović et al., 2012; Kilinç et al., 2012; König and Rothland, 2012; Lin et al., 2012; Watt and Richardson, 2012). These results suggest that teachers from different subjects might share similar types of motivation to enter the teaching profession.

Second, the ratings of native and non-native preservice CSL/CFL teachers on the importance of four types of motivation were similar. The ratings of the two groups on altruistic, cross-cultural, and intrinsic values were significantly higher than the midpoint of 3, and the effect sizes were large. Although the two groups differed significantly in the ratings of cross-cultural and intrinsic values, the effect size was small. Moreover, the ratings of both the groups on fallback career choice were not significantly different from the midpoint of 3 or from each other and had small effect sizes. These results suggest that the contribution of these four types of motivation to the choice of native and non-native preservice CSL/CFL teachers regarding a teaching career might be similar.

#### Cross-Cultural Value

Cross-cultural value relates to the contribution of interest in cross-cultural exchange and communication to the decisions of the individuals to enter the teaching profession. This finding was in line with the previous findings (Zhu and Qian, 2015; Zhang et al., 2020) and indicates that L2 teachers might have a unique type of motivation to enter the language teaching profession that has not been reported among teachers in other subjects. Cross-cultural value might be closely related to intrinsic value or extrinsic value, yet it emerged as an independent factor in both the two groups, suggesting its uniqueness as a specific type of motivation. Similarly, in the interview, the common reasons for choosing MTCSOL program the participants mentioned included, “interest in foreign cultures,” “liking exploring different cultures,” “the international context of Chinese teaching,” or “interest in China and Chinese cultures.”

The next question to answer is why the two groups had such a strong cross-cultural value. For the native group, as suggested by the model of Borg (2003) about the factors influencing teacher cognition, their previous experiences in learning foreign languages or interacting with foreigners and the general desire to understand foreign cultures in the Chinese society, together along with the prospective interaction opportunities with foreigners CSL/CFL teaching could provide, may collectively contribute to their strong cross-cultural value. As for the non-native group, interest in L2 culture or cross-cultural communication was a major type of L2 learning motivation (Gardner, 1985; Williams and Burden, 1997); however, they could encounter different challenges coming from various aspects in China, such as teacher-centered instruction and mismatch in assumed benefits of Chinese-only principle between CSL/CFL teachers and learners inside the classroom, and Chinese lifestyles outside the classroom (Gong et al., 2020b). Their identity as language learners or users in China as well as their desire to overcome the challenges they faced urged them to adopt various strategies to learn about cross-culture knowledge for cultural adaption (Gong et al., 2020b, 2021) to “become sufficiently engaged in local communicative settings” (Kinginger, 2013, p. 342). More importantly, the strong cross-cultural motivation of the two groups was in line with intercultural communicative competence, which has been set as a key goal in the second language teaching (Byram, 1997; Kusumaningputri and Widodo, 2018) and similarly emphasized in the *Standards for Teachers of Chinese to Speakers of Other Languages* (Hanban, 2007). The cross-cultural motivation of the two groups might help fulfill their role in developing the intercultural communicative competence of CSL/CFL learners. However, these explanations are tentative and more studies are needed in the future.

#### Intrinsic and Altruistic Values

Intrinsic value was strongly emphasized in the expectancy-value model (Eccles, 2005), and it was also similarly highly rated by the non-native and native teachers in the present study. For instance, all the four native Chinese participants interviewed believed that they had suitable abilities for teaching and liked “teaching Chinese to foreigners” or “teaching languages, such as Chinese and English.” Similarly, a Russian participant mentioned that, “the status of teacher in Russia was very low; [they are] treated just like waiters, yet I still like teaching and want to become a teacher.”

The high ratings of the participants on the importance of the altruistic value of teaching in the present study were largely in line with the metaphors of the preservice CSL/CFL teachers (Ma and Gao, 2017), who described CSL/CFL teachers as a “dandelion that flies across the ocean” and “miniature of China.” These results were consistent with the above-midpoint ratings for shaping the future, making social contributions and enhancing social equity which have been reported in studies using the FIT-Choice scale across different cultural backgrounds, such as Turkey, China, the US, the Netherlands, and Croatia (Watt and Richardson, 2012). For example, some participants mentioned that “I can alleviate foreigners' stereotypes about China and Chinese people by teaching Chinese” and “Helping my students learn about China and Chinese cultures is a success for me.” However, this finding was inconsistent with the research by Shih (2016), which reported that social contribution was more frequently mentioned by the native English teachers than the local Taiwanese teachers. This difference might be related to the qualitative method and limited number of participants (*n* = 38) in the study by Shih. These conflicting results indicate that further studies are needed to explore this issue, such as the sources of the altruistic value of CSL/CFL teachers (Ma and Gao, 2017; Gong et al., 2018a).

#### Fallback Career Choice

The results of the present study indicate that the native and non-native pre-service CSL/CFL teachers were unlikely to choose teaching as a fallback career choice or as a stepping stone to another career. This finding was consistent with previous studies using the FIT-Choice scale, which reported that the rating of fallback career choice was the lowest among teachers in both developed and developing countries (Watt and Richardson, 2012). However, it cannot be guaranteed that all the recruited participants provided homogeneously low ratings of fallback career choice. In fact, in the present study, 14.77% of the native participants (*n* = 48) and 17.54% of the non-native participants (*n* = 57) had ratings of fallback career choice higher than 4 (*important*). These results suggest that a small proportion of the participants might have failed to enter or pursue their first-choice career and demonstrated a less positive motivational profile. In the interviews, some participants said, “I was not sure about the most suitable job for me after graduation, so I will teach Chinese first to see whether it fits me,” “Teaching Chinese is not my ideal job” and “I did not know what to do after graduation and chose to study in the MTCSOL program to explore the real China.” However, whether and how the motivation of the participants pursuing changes in fallback career choices is not clear and requires further research.

### Differences in the Motivation Between Native and Non-native CSL/CFL Teachers

The native and non-native pre-service CSL/CFL teachers differed significantly in the extrinsic value and social influence, where the non-native groups significantly scored higher than the native group. These results suggest that the non-native group might be motivated more by extrinsic value and social influence than their native counterparts to enter CSL/CFL teaching, which could be accounted for by the difference between the two groups in cultural and teaching contexts.

For most of the non-native preservice CSL/CFL teachers, teaching Chinese is a domestic job in terms of teaching contexts. In most countries, working at schools or universities is generally a stable and respected job, which is widely considered as very attractive for the females, who dominate the non-native preservice CSL/CFL teachers. The salaries offered by Chinese teaching are likely to be competitive because the increasing economic and cultural links between China and foreign countries yield continuing demand for highly proficient CSL/CFL speakers, whose number is yet limited (Ruan et al., 2016; Wang, 2018). In terms of cultural contexts, most of the non-native participants came from Asia, where family ties are strongly valued, and their career choice might be heavily influenced by family members or friends. This account could be corroborated by the interview results, such as “Chinese teaching is a stable job,” “Teaching Chinese is highly respected and high-salary [in Korea]” and “Most of my family members are teachers, so teaching is an obvious and stable choice for me.”

As for the native group, *going abroad* culture and teaching Chinese as an international job might be the main reasons. Just as a traditional Chinese saying goes, read 10,000 books and travel 10,000 miles (读万卷书, 行万里路), working or studying abroad has been encouraged by the Chinese people in modern societies. The total number of students studying abroad reached 6.56 million from 1978 to 2019 (Ministry of Education of the People's Republic of China, 2020) and Chinese students are ranking the top among the international students in the developed countries (Henze and Zhu, 2012; Thøgersen, 2016; Chao et al., 2017; Universities UK, 2018). However, going abroad, in particular to developed countries, is expensive and poses a great challenge for the middle and lower classes. Meanwhile, Competitive Local Exchange Carriers (CLEC) has been funding thousands of CSL/CFL teachers to work abroad every year in the recent decade. Thus, being a CSL/CFL teacher could be a good choice for students from the middle and lower classes who desire to go abroad, and this unique experience is of great value for job hunting and career development in China (Zhu and Qian, 2015). Therefore, the native preservice CSL/CFL teachers might value the potential experience of working abroad more than salary or stability. In addition, teaching Chinese abroad is international in nature and mainly managed by CLEC and Confucius Institutes/Classrooms, where the influence from the family members or friends of the applicants is minimal. This assumption is consistent with the comments of the participants, such as that, “the experience of teaching Chinese in Ireland might make my resume eye-catching,” “teaching Chinese could help you learn more about everything and be helpful for future jobs,” and “Although my parents have traditionally considered teaching to be an ‘iron rice bowl’ job (a job with guaranteed job security), their perceptions of CSL/CFL teaching is limited or distorted.”

In summary, the overall results of the present study contribute some new knowledge to the area of CSL/CFL teaching, in particular, to the education of preservice CSL/CFL teachers. One major contribution is that, in contrast to the commonly held assumption that native and non-native preservice CSL/CFL teachers might differ a great deal in teaching-related factors, the two groups were found to have similar motivations for entering the CSL/CFL teaching profession and had similar ratings of the importance of four of the six factors, with significant differences observed only in the external factors, such as extrinsic motivation and social influence. These results suggest that native and non-native preservice CSL/CFL teachers might share commonalities in other internal aspects, such as teacher cognition or meta-cognition of teaching abilities. This new finding could be significant for the design of MTCSOL programs, which is discussed in section Implications.

### Implications

#### Theoretical Implications

In terms of theoretical significance, the results of the present study confirm that teachers of different subjects have some similar types of motivations (Dörnyei and Ushioda, 2011; Watt and Richardson, 2012; Han and Yin, 2016), for example, placing strong importance on the intrinsic and altruistic values. However, the finding about cross-cultural value in native and non-native preservice CSL/CFL teachers sheds light on the uniqueness of L2 teacher motivation, which might relate to the influence of subject specialization (Watt and Richardson, 2012). In addition, the differences between native and non-native groups in extrinsic value and social influence point to the potential influence of socio-cultural settings and teaching contexts on teacher motivation. Based on the overall findings of the present study and implied by the ecological system theory (Bronfenbrenner, 1977, 1986; Bronfenbrenner and Evans, 2000; Hornberger, 2003; Lier, 2004; Kramsch, 2008; Steffensen and Kramsch, 2017; Mohammadabadi et al., 2019), teacher motivation might be jointly influenced by variables at different levels, such as native language background at the microsystem, subject specialization at the mesosystem, teaching context at the exosystem, and social cultures at the macrosystem. That is to say, teacher motivation might not be “a product of a limited number of factors, but it is a constellation of various distal and proximal factors to the teachers' classes” (Mohammadabadi et al., 2019, p. 765). However, this account is tentative, and more supporting evidence will be needed from future studies.

In addition, the present study further corroborates the validity and reliability of the FIT-Choice scale in exploring the motivation of the language teacher (Shih, 2016; Kissau et al., 2019a,b; Zhang et al., 2020) by providing evidence from both native and non-native pre-service CSL/CFL teachers. The general results provide further support for the application of expectancy-value theory in exploring second language teacher motivation.

#### Practical Implications

In terms of practical implications, the findings of the present study might be useful to help solve the problem of CSL/CFL teacher shortage outside China. The solution lies in attracting preservice CSL/CFL teachers and retaining in-service CSL/CFL teachers, whose motivation should be taken into account in designing MTCSOL program and administration of in-service teachers.

In terms of designing MTCSOL program, the findings of the present study suggest the potential application of a mixed-class model for native and non-native preservice CSL/CFL teachers. A noteworthy trend in teacher education is to view teachers as agents in knowledge-building practice and to provide chances for teachers to take part in this practice by forming a community, where they can share, discuss, improve, and transfer ideas (Darling-Hammond and McLaughlin, 1995; Sachs, 2005; Hong and Sullivan, 2009; Yang, 2021). A mixed-class model, where the two groups study together and form a teaching community to some extent, could align with the teacher motivation of the two groups.

The mixed-class model could boost the cross-cultural value of the two groups. The study found that native CSL/CFL teachers were limited in intercultural communicative competence (Gong et al., 2018a), which is yet emphasized in *Standards for Teachers of Chinese to Speakers of Other Languages* (Hanban, 2007). Meanwhile, the identity of non-native preservice CSL/CFL teachers as language learner/user/teacher could drive them to undertake Chinese language and culture learning (Gong et al., 2021), as an investment to gain access to various symbolic and material resources (Li and Li, 2020). Thus, the mixed-class model as a teaching community could serve as a platform to meet the needs of both the groups, where the two groups demonstrate and learn different cultures, enrich cross-cultural knowledge, and develop intercultural communication competence.

The mixed-class model could also benefit the intrinsic motivation of the two groups. In the teaching community of the mixed-class model, the interactions with native Chinese speakers could facilitate the development of the Chinese language proficiency of the non-native groups and their confidence in Chinese teaching, considering that anxieties caused by self-perceived insufficient L2 proficiency might influence language teaching (Horwitz, 1996; Lee et al., 2017; Calafato, 2019). For the native group, the mixed-class model could mimic different CSL/CFL teaching contexts and deepen their understanding of Chinese language acquisition, which further contributes to the growth of teaching skills, similar to the effect of practicum on the growth of teaching competence (Yuan and Lee, 2014; Aghabarari and Rahimi, 2020; Qiu et al., 2021). More importantly, most native CSL/CFL teachers tend to undergo a transformation in pedagogical beliefs and practices when working abroad due to the different educational contexts in China and other countries (Moloney, 2013; Wang and Du, 2014; Moloney and Xu, 2015). This model could provide opportunities for the native group to experience the different education settings in advance and alleviate the troubles caused by the transformation.

In sum, the mixed-class model could be viewed as a response to the call of the researchers for integrating cross-cultural elements into teacher education programs to fulfill the professional roles of CSL/CFL teachers (Moloney, 2013; Lai et al., 2015; Moloney and Xu, 2015; Jin and Dervin, 2017; Gong et al., 2018a). These assumed benefits of the mixed-class model could strengthen the motivations of the two groups and thus might attract them to enter the Chinese teaching profession. However, this suggestion is tentative, and the details of a mixed-class model need to be carefully designed in future research.

As for retaining in-service CSL/CFL teachers, measures are recommended to be taken against demotivation. In the context of L2 teaching, demotivation concerns external or internal factors that negatively affect the willingness of an individual to teach. Researchers have identified different categories of variables that might lead to demotivation, such as students, stress, inhibition of teacher autonomy, and limited potential for intellectual development (Kiziltepe, 2008; Sugino, 2010; Dörnyei and Ushioda, 2011; Yaghoubinejad et al., 2017). Policymakers in CSL/CFL education organizations or institutions are advised to tackle the influencing factors that might demotivate the in-service teachers in different contexts and take necessary measures to create an environment that could retain them for CSL/CFL teaching, such as improving the welfare and stability of Chinese teaching. Due to the limited scope of the present study, the measures to retain in-service teachers are not discussed in detail here.

## Conclusion

The present study explored the types of teacher motivation and the ratings of the importance of each type of motivation between native and non-native preservice CSL/CFL teachers. The results highlight that the two groups had more similarities than differences in the types and ratings of teacher motivation, providing a new perspective in comparing the native and non-native L2 teachers. The findings further suggest the uniqueness of cross-cultural value in L2 teacher motivation in comparison to that in general education and the valid application of FIT-Choice scale in exploring teacher motivation in the contexts of teaching Chinese.

The present study was subjected to several limitations, which could be overcome in future research. The first limitation concerns the language in which the questionnaire was presented to the non-native group. Although the recruited non-native participants were at an advanced level of Chinese and a pilot study was conducted before the formal data collection, the participants might have had different results if the instructions and statements had been presented in their native languages. Therefore, developing valid and reliable multilingual versions of CSL/CFL teacher motivation questionnaires that are comparable with the Chinese version is one future research direction.

The second limitation lies in the representativeness of the non-native sample. Snowball sampling method was used in the present study, and most of the recruited non-native CSL/CFL participants came from Asia, such as Thailand and Vietnam, and were registered at universities in Mainland China. Therefore, the results generated from the present study might not be generalizable to teachers from other areas, such as Europe and America, or those who study in their home countries. Considering the potential influence of cultural and socioeconomic factors on teacher motivation (Watt and Richardson, 2012), comparing teacher motivation among pre-service CSL/CFL teachers from different countries might reveal more insightful findings about this topic.

Third, the present study mainly depended on a questionnaire to explore the motivations of pre-service CSL/CFL teachers, and the interview results were supplementary. Considering the pros and cons of quantitative and qualitative research, qualitative studies using in-depth interviews or narrative research could be carried out in the future to comprehensively understand the underlying reasons accounting for the similarities and differences in teacher motivation among native and non-native CSL/CFL teachers.

Fourth, it has been commonly acknowledged that motivation is likely to change over time (Richardson et al., 2014; Han and Yin, 2016); however, the present study only explored the motivation of native and non-native CSL/CFL teachers in the preservice stage. Therefore, future research could investigate the change path of teacher motivation from preservice to in-service stage among second language teachers.

Despite the above-mentioned limitations, the findings of the present study showcased the significance of understanding teacher motivation in the context of CSL/CFL teaching. Meanwhile, we call for more attention to the design of MTCSOL program based on the empirical studies on CSL/CFL teacher education to further mitigate the problem of the shortage of CSL/CFL teachers outside China.

## Data Availability Statement

The raw data supporting the conclusions of this article will be made available by the authors, without undue reservation.

## Ethics Statement

The studies involving human participants were reviewed and approved by Ethics Committee in College of International Education, Minzu University of China. The patients/participants provided their written informed consent to participate in this study.

## Author Contributions

LG was responsible for research design and article drafting. BW was responsible for research design, data collection, and data analysis. HZ was responsible for research design, data collection, data analysis, and article drafting. All authors contributed to the article and approved the submitted version.

## Conflict of Interest

The authors declare that the research was conducted in the absence of any commercial or financial relationships that could be construed as a potential conflict of interest.

## Publisher's Note

All claims expressed in this article are solely those of the authors and do not necessarily represent those of their affiliated organizations, or those of the publisher, the editors and the reviewers. Any product that may be evaluated in this article, or claim that may be made by its manufacturer, is not guaranteed or endorsed by the publisher.

## Footnotes

^1^The data were cited from the news report released by International Chinese Language Education Conference (2019). http://conference2019.hanban.org/page/#/pcpage/detailpage/newsdetail?id=42.

^2^The data were cited from the official website for Master's degree programs in China. https://yz.chsi.com.cn/zsml/queryAction.do.

^3^The data were cited from a personal communication with an official in CLEC.

^4^Hanyu Shuiping Kaoshi (HSK) is a standardized Chinese language proficiency test. It has six levels, with level 1 as the beginner level and level 6 as the advanced level. According to the Center for Language Education and Cooperation, the minimum Chinese language proficiency for non-native Chinese speakers to register in MTCSOL program is level 5 (https://cis.chinese.cn/Account/Proceduresfor).

^5^The interview was mainly used to design the questionnaire in the present study, instead of a key research instrument in collecting data on teacher motivation. According to the comments of a reviewer, some of the interview results were mentioned in the Discussion section to support the findings generated from the questionnaire survey.

^6^The commonly used cut-off factor loading is 0.30 or 0.40 in the areas of psychological research and language education. We first used 0.30 and found that some items were loaded on two factors; therefore, the cut-off factor loading was changed to 0.40 to make the results more reasonable.
