# Supplementary material for: A Comparative Study of the Motivations to Teach Chinese Between Native and Non-native Pre-service CSL/CFL Teachers
Source: Front Psychol. 2021 Aug 17;12:703987. doi: 10.3389/fpsyg.2021.703987 (PMC8416099; doi:10.3389/fpsyg.2021.703987)
Supplement: Supplementary file 1 [file Data_Sheet_1.docx]

# **Appendix**

Table 1 Summary of Language Teacher Motivation Research

| Article | Participants | Method | Main results |
| --- | --- | --- | --- |
| Pennington,  1995 | 32 in-service ESL teachers in US and other countries;  native and nonnative teachers | questionnaire | 1. Highest level of self-reported job satisfaction occurs in internal rewards involving service and moral values. 2. Least satisfied with external rewards, advancement and financial compensation, and policies and practices. |
| Kyriacou & Benmansour,  1999 | 83 ESL teachers in Morocco 69,  French as L2 in UK;  nonnative teachers |  | 1. Top reasons for teaching: I enjoy the subject I will teach; I want to help children succeed; The language is important to me; I like the activity of classroom teaching. 2. Morocco > UK Being a teacher can lead on to other jobs in the future; It enables me to use the foreign language; My experiences as a pupil gave me a positive image of the job; It is a socially well regarded job. 3. UK>Morocco I like working with children; I can get a job in any part of the country. |
| Kassabgy et al., 2001 | 107 experienced ESL teachers in Egypt (70) and Hawaii (37);  native and nonnative teachers | questionnaire | 1. Values most emphasized: helping students to learn, perform to the best of one's ability. 2. Intrinsic value over extrinsic values such as salary, title and opportunities for promotion |
| Barnes,  2005 | 235 L2 pre-service teachers in UK | questionnaire | Reasons to teach MFL: love of languages, the enjoyment of languages, the crucial nature of the cultural aspects of the subject, and the pre-eminence of communication |
| Zhao, 2008 | 17 secondary ESL teachers at 5 secondary schools in mainland China;  nonnative teachers | life-history narrative | 1. By default: the demand of the job market, provisional, accident, arrangement 2. Enjoying the subject: love of English language and culture, enriching identity and life experience, possibility for other jobs 3. Job security: safe way to make a living, professional fulfilment, ongoing learning 4. Having influence on people: nurturing students to learn and grow, ideals and desires to advance the system |
| Karavas,  2010 | 224 in-service ESL teachers in Greece;  nonnative teachers | questionnaire | 1. Top five reasons for entering teaching profession: working with young people, love of the subject, mentally stimulating work, job security, changing students' lives/attitudes 2. Least rated reasons: salary and benefits, status of the profession, family approval |
| Erkaya,  2012 | Eight in-service ESL teachers at universities in Turkey; nonnative teachers | interview | 1. Intrinsic: wanting to become a teacher, being born to be teacher 2. Extrinsic: working with students, teaching at university |
| Shih,  2016 | 38 in-service ESL teachers in Taiwan;  native and nonnative teachers | interview | 1. Intrinsic value: enjoy English or wanted to become a teacher since childhood 2. Extrinsic value: competitive salary, balance between work and life, health 3. Prior teaching experience 4. Social influences and social dissuasion, being a teacher could be a family heritage. 5. Local teacher rarely mentioned social contribution |
| Kissau, Davin & Wang, 2019 | 54 pre-service ESL teachers in US;  native and nonnative teachers | questionnaire + interview | 1. Top reasons for teaching: love of the language, perceived social contribution, the ability to shape the future of children, perceived ability to teach, prior teaching and learning experience 2. Least mentioned reasons: time for family, social influences, fallback career |
| Kissau et al, 2019 | 54 American, 233 German and 116 Chinese pre-service L2 teachers;  native and nonnative teachers | questionnaire + interview | Commonly highly rated values: love of the language, shape future of children/adolescents, social contribution, work with children and adolescents, intrinsic career value, teaching ability |
| Author (2020) | 411 pre-service CSL/CFL teachers in mainland China;  native teachers | questionnaire | 1. Values with high ratings: cross-culture value, intrinsic value, altruistic value 2. Values with low ratings: extrinsic value, fallback career, social influences |

Table 2. Questionnaire items for native and nonnative pre-service CSL/CFL teachers after factor analysis

| Factor | Items for native group | Items for nonnative group |
| --- | --- | --- |
| Cross-  cultural  Value | 1. Chinese teaching could allow more family time. 2. I like working in an environment that involves being in contact with foreigners#. 3. I have had pleasant communication experiences with foreigners#. 4. As a CSL/CFL teacher, I can have more opportunities to work abroad. 5. I am interested in learning about different cultures. 6. I like teaching others to learn a foreign language. | 1. I have had pleasant communication experiences with Chinese people#. 2. I like working in an environment that involves being in contact with Chinese people#. 3. I like socializing with Chinese people. |
| Intrinsic  Value | 1. Teaching is a career suited to my abilities#. 2. I am interested in teaching#. 3. I have good teaching skills#. 4. I have the qualities of a good teacher#. 5. I have always wanted to be a teacher#. | 1. Teaching is a career suited to my abilities#. 2. I am interested in teaching#. 3. I have good teaching skills#. 4. I have the qualities of a good teacher#. 5. I have always wanted to be a teacher#. |
| Extrinsic  Value | 1. As a CSL/CFL teacher, I can have a stable income#. 2. As a CSL/CFL teacher, I can have a high salary#. 3. I like socializing with foreigners. 4. Chinese teaching is a secure job#. | 1. As a CSL/CFL teacher, I can have a stable income#. 2. As a CSL/CFL teacher, I can have a high salary#. 3. As a CSL/CFL teacher, I can have more opportunities to work abroad. 4. Chinese teaching is a secure job#. |
| Social  Influence | 1. My friends think I should be a CSL/CFL teacher#. 2. My teachers/classmates think I should be a CSL/CFL teacher#. 3. My family/relatives think I should be a CSL/CFL teacher#. | 1. My friends think I should be a CSL/CFL teacher#. 2. My teachers/classmates think I should be a CSL/CFL teacher#. 3. My family/relatives think I should be a CSL/CFL teacher#. |
| Altruistic  Value | 1. Chinese teaching can help improve China’s international image. 2. Chinese teaching can help communicate Chinese culture to other countries. 3. Chinese teaching can help eliminate foreigners’ misunderstandings of China. | 1. Chinese teaching can help communicate Chinese culture to my country. 2. Chinese teaching can help my compatriots have a better understanding of China. 3. Chinese teaching can help my compatriots learn Chinese. |
| Fallback  career Value | 1. I have not found my ideal major yet#. 2. I chose CSL/CFL teaching as a last-resort career#. 3. I was unsure of what career I wanted#. | 1. I have not found my ideal major yet#. 2. I was unsure of what career I wanted#. 3. I chose Chinese teaching as a last-resort career#. |
| Removed items | NA | 1. I like teaching others to learn a foreign language. 2. Chinese teaching could allow more family time. 3. I am interested in learning about different cultures. |

# Items used for MANOVA tests.
